# Management of Noncatastrophic Internal Carotid Artery Injury in Endoscopic Skull Base Surgery

**DOI:** 10.7759/cureus.5537

**Published:** 2019-08-30

**Authors:** Michael Safaee, Jacob S Young, Ivan H El-Sayed, Philip V Theodosopoulos

**Affiliations:** 1 Neurological Surgery, University of California, San Francisco, USA; 2 Otolaryngology Head and Neck Surgery, University of California, San Francisco, USA

**Keywords:** skull base surgery, endoscopic endonasal surgery, internal carotid artery injury

## Abstract

Arterial injuries are the most feared complication of endoscopic skull base surgery. During resection of the middle fossa component of a large ventral skull base chondrosarcoma, arterial bleeding was encountered near the right internal carotid artery (ICA). Durable hemostasis could not be achieved with packing and the patient was taken for an emergent angiogram that revealed a pseudoaneurysm of the proximal intradural ICA. Given the presence of good collateral flow through the anterior and posterior communicating arteries, the right ICA was sacrificed by coil embolization. The patient was taken back to the operating room for closure then transferred to the intensive care unit and maintained on vasopressors for five days to ensure adequate perfusion. The right ICA was coil embolized and the patient was taken back to the operating room for closure. The patient recovered without complication. Arterial injuries, although serious, are not always catastrophic. Critical steps are immediate recognition of bleeding, vascular imaging, and vessel sacrifice if necessary.

## Introduction

Endoscopic endonasal approaches provide exceptional views and access to the skull base and are increasingly popular for the resection of pituitary tumors and ventral skull base lesions. Although rare, arterial injuries are among the most feared complication with rates ranging from 0.1% to 0.9% [1-8]. Although the endoscope expands visualization, this dramatic improvement in exposure compared to traditional approaches leads to an increase in the complexity of cases where normal anatomic landmarks are distorted. In certain cases, the endoscopic approach can preclude obtaining proximal control of the offending vascular injury and due to anatomic and technical constraints.

Preferred techniques for the management of arterial injury during endoscopic surgery are a topic of debate and include direct cautery, clip ligation, tamponade with muscle or hemostatic agent, and endovascular treatments including occlusion, coiling, or stenting [1-5, 7-9]. There are published algorithms for the use of stenting, coiling, and internal carotid artery (ICA) sacrifice for the treatment of pseudoaneurysms or active extravasation, however, there are no consensus guidelines [10]. We present the case of a noncatastrophic ICA injury during endoscopic resection of a complex skull base chondrosarcoma that highlights several key principles including the need for prompt vascular imaging and viability of carotid sacrifice.

## Case presentation

**Figure 1:**
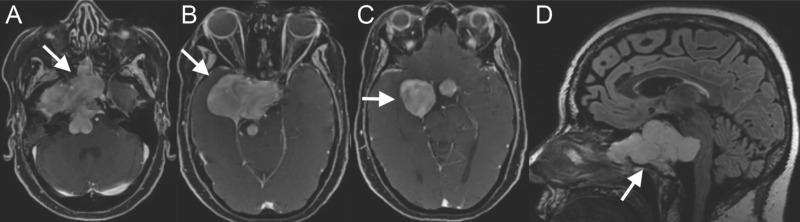
Preoperative MRI on large skull base chondrosarcoma. Axial T1-weighted MRI with contrast showed a large 6.9 cm (anterior-posterior) x 6.6 cm (lateral) x 6.9 cm (craniocaudal) contrast-enhancing lesion centered in the right petroclival synchondrosis extending into the right middle cranial fossa, cavernous sinus, sella, sphenoid sinus, and nasopharynx (A, B, and C). Sagittal FLAIR MRI illustrates the craniocaudal extent of the lesion with brainstem compression (D).

A 55-year-old woman presented with several months of diplopia and complete ophthalmoplegia of the right eye. MRI revealed a 6.9 cm x 6.6 cm x 6.9 cm destructive lesion involving the sphenoid bone and extending into the sphenoid sinus, cavernous sinus, suprasellar cistern, and posterior fossa most consistent with a chondrosarcoma (Figure 1).

The patient was taken to the operating room for an endoscopic endonasal resection of this lesion and provided informed consent for use of her case for academic and research purposes. A lumbar drain was placed preoperatively and kept clamped throughout the procedure. An expanded endoscopic approach was performed with wide maxillary antrostomy and ethmoidectomy. A nasal septal flap was harvested and rotated into the maxillary sinus. The sphenoid sinus was opened and tumor was mobilized away from the sinus and dissected from the sella. Tumor was followed inferior and dissected off the clivus and superiorly off the suprasellar arachnoid. After tumor mobilization and debulking, the dura of the clivus was noted to be incompetent and opened to allow for mobilization of tumor from the arachnoid of the posterior fossa. Attention was then turned to debulking tumor from the lateral gutter of the sella, cavernous sinus, and middle fossa with the use of angled endoscopes and instruments. At the conclusion of the resection, persistent noncatastrophic arterial bleeding was noted near the right ICA, behind which the most lateral dissection had been performed in an attempt to resect the middle fossa component. No clear point of extravasation was identified, however, the ICA was not fully exposed. Although the bleeding could be controlled with direct focal packing of hemostatic material, there was no way to provide durable hemostasis and thus the area was packed and the patient was taken for an emergent angiogram intending to come back to the operating room for completion of the closure.

**Figure 2:**
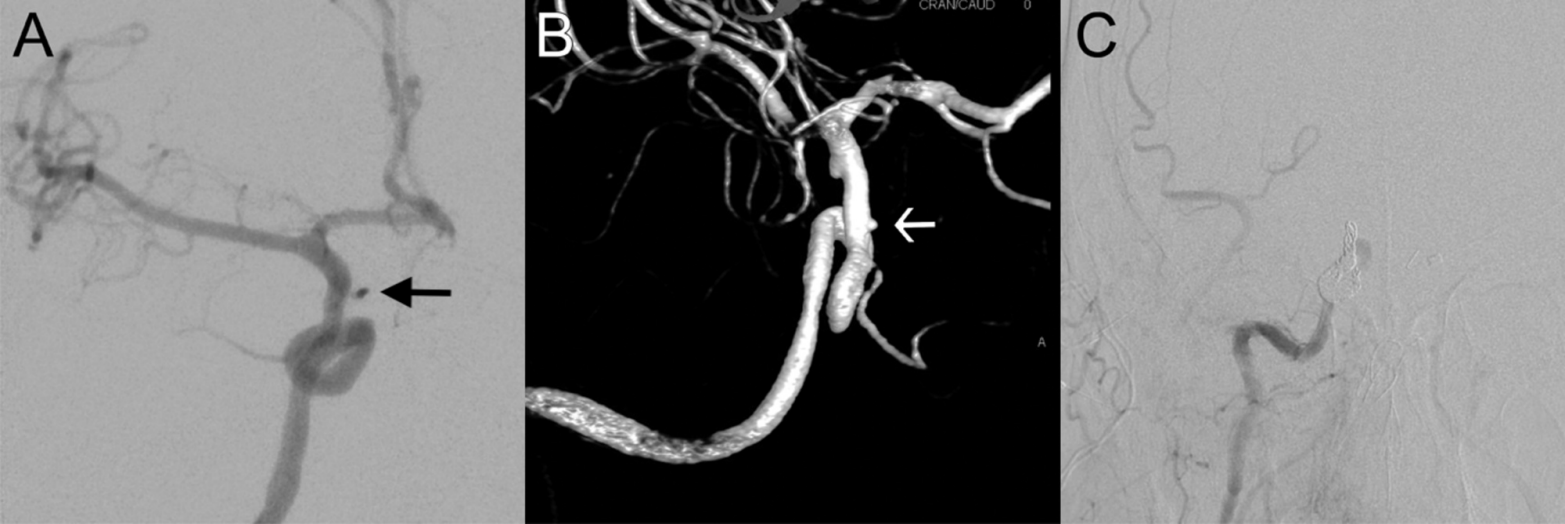
Angiogram demonstrating internal carotid artery (ICA) injury. Anterior-posterior projection demonstrates a small pseudoaneurysm along a branch arising from the right ICA proximal to the intradural segment (A), also shown on a three-dimensional reconstruction (B). Given the presence of good collateral flow, a decision was made to proceed with coil embolization of the right ICA at the ophthalmic segment (C).

The angiogram showed a small pseudoaneurysm along a branch arising from the ICA likely proximal to the intradural segment although the exact branch was difficult to identify as the course of the ICA had been significantly altered by the presence of the tumor (Figure 2A,B). Given good collateral flow through the anterior communicating artery and posterior communicating artery (PCom), a decision was made to proceed with carotid sacrifice with coil embolization of the right ICA at the ophthalmic segment (Figure 2C). Subsequent angiographic runs confirmed filling of the ophthalmic branch through ethmoidal branches of the external carotid artery.

**Figure 3:**
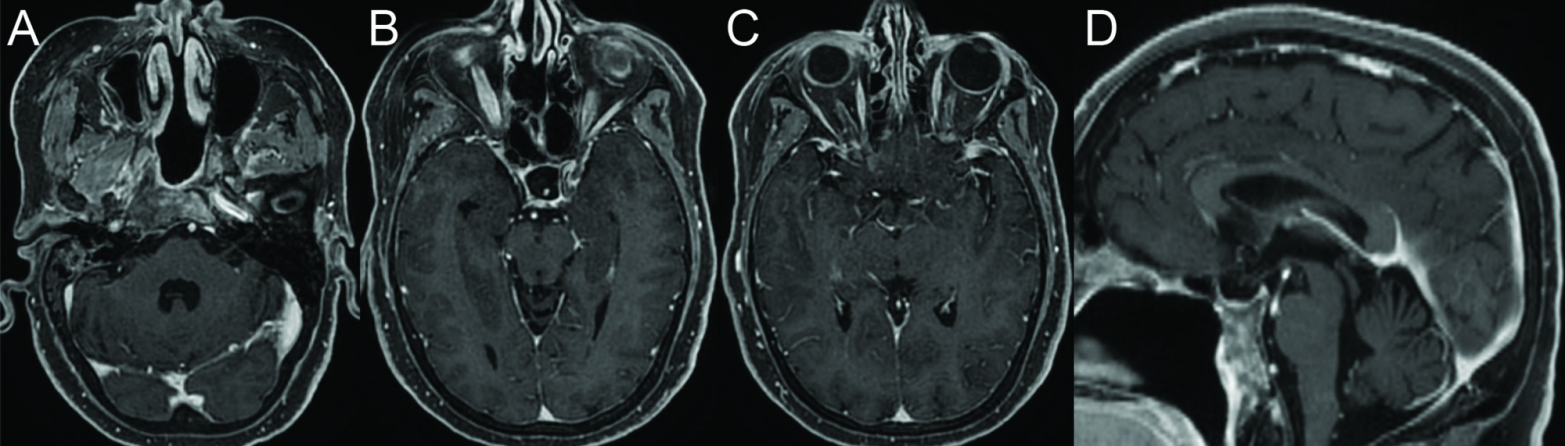
Postoperative MRI after resection of skull base chondrosarcoma. Axial T1-weighted MRI with contrast shows near total resection of the chondrosarcoma two years after surgical resection (A, B, and C). Sagittal MRI shows dramatic reduction in tumor size and alleviation of brainstem compression (D).

The patient was transferred back to the operating room where the nasal packing and hemostatic material was removed with gentle irrigation. The sella was filled with an abdominal fat graft and the wound closed with the previously harvested nasal septal flap and the nose packed with NasoPore (Stryker, Kalamazoo, MI, USA) and silastic splints. The patient was transferred to the intensive care unit and maintained on vasopressors for five days to ensure adequate perfusion. Postoperative MRI showed near total resection with a small residual along the most anterior and lateral aspect of the middle fossa tumor component. Final pathology was chondrosarcoma, WHO grade II. She was discharged home in good condition and subsequently underwent fractionated radiotherapy with intensity-modulated radiation therapy (IMRT) for a total of 66 Gy in 33 fractions. She also underwent strabismus surgery to help accommodate for her ophthalmoplegia and on her last clinic visit two years after surgery was doing well with stable tumor on imaging and resolved cranial nerve deficits (Figure 3).

## Discussion

We present a noncatastrophic ICA injury during an endoscopic endonasal approach to a complex skull base chondrosarcoma. Large tumors are at increased risk for carotid injury due to the distortion of anatomic landmarks. This case represents a minor arterial injury, likely a cavernous ICA branch, and shows that not all ICA injuries result in catastrophic hemorrhage. Regardless of magnitude, the key is to recognize that the source is arterial, obtain immediate imaging, and obtain hemostasis in a permanent fashion with vessel sacrifice if necessary. This is the fourth arterial injury that the senior author has encountered in more than 1,000 endoscopic cases and our opinions here are reflective of that limited experience with this event. 

Arterial injuries occur in just 0.1%-0.9% of endonasal endoscopic skull base surgery [1-8]. ICA injuries are even more rare, occurring in 0%-0.26% of cases [8]; they can also present in a delayed fashion with the development of pseudoaneurysms or delayed hemorrhage [11]. Reported rates of arterial injury are likely an underestimation of the real incidence as most such injuries go unreported. The rate of ICA injury is not significantly higher in endoscopic skull base surgery when compared to a large contemporary series of microscopic endonasal transsphenoidal pituitary surgeries performed between 2001 and 2010; among 70,878 patients, the rate of intracerebral hemorrhage or subarachnoid hemorrhage was 0.9% [9]. Although the complexity of endoscopic skull base tumor surgeries has undoubtedly increased, increased visibility provided by the endoscope and use of intraoperative navigation likely result in a safer operation. 

The management of arterial bleeding starts with visualization of the offending vessel and assessment of the injury. Catastrophic injuries of the ICA are immediately life threatening and can acutely obscure the field of vision. Techniques such as bipolar coagulation, vessel ligation, and direct placement of hemostatic material on the vessel tear or hole are options that few can perform safely and reproducibly [1-3, 8-9]. Any aggressive move, particularly direct manipulation of the injured vessel, can worsen the injury. The most common and safest maneuver is surgical packing, which although not permanent, is an effective temporizing measure that allows for vascular imaging and definitive management, even in cases of noncatastrophic hemorrhage. We caution against the use of hemostatic material without vascular imaging, even if complete hemostasis may be obvious, as subsequent dislodging of the hemostatic clot can result in uncontrolled extravasation or the development of a pseudoaneurysm which are very morbid and possibly avoid complications. Based on the trauma literature, pseudoaneurysms can cause stroke in 33% of patients and carry a mortality rate of over 10% [12].

Romero et al. reported their experience with four arterial injuries in 800 cases (0.5%), among which two were treated with coil occlusion, one with coil embolization, and one with Weck clip occlusion [8]. The authors also identified eight studies of at least 100 patients that included 7,336 patients with 25 arterial injuries (0.34%), 19 of which involved the ICA (0.26%). Sylvester et al. presented their individual experience in 576 transsphenoidal pituitary adenoma surgeries and found seven cases of ICA injury (0.1%) [13]. All were managed with endovascular techniques: three ICA sacrifice with coil placement, two coil or stent-assisted coilings, and two endoluminar reconstruction with flow diversion. In their review of the literature for endovascular-based therapies of intraoperative ICA injuries (pseudoaneurysms, active hemorrhage, carotid-cavernous fistula (CCF), stenosis, and dissection), they found that ICA sacrifice was the most common treatment (43.8%) followed by endoluminar reconstruction (29.5%), and lesion embolization (26.7%). Direct vessel repair or clip reconstruction is typically not feasible when carotid exposure is limited. Patients who underwent ICA sacrifice had new neurologic deficits in 28% of cases, 22% of which were permanent. Among published cases of coil embolization, successful treatment was observed in 57% of cases. Flow diversion is an emerging therapy for pseudoaneurysm repair with a success rate of 58%.

Zhang et al. proposed an algorithm for the management of iatrogenic ICA injuries that utilizes covered stents and hybrid operating rooms, both of which have increased the rate of vessel preservation after injury [14]. This algorithm includes a delayed angiogram when nasal packing is removed to allow for a rapid response to any catastrophic rebleeding. While covered stents allow for vessel preservation, they also require antiplatelet and anticoagulant therapy, which increases the risk for delayed hemorrhage in the surgical field. When the parent artery is not suitable for a covered graft and must be occluded, an external carotid to internal carotid bypass is only necessary when there is poor collateral circulation.

Other important factors include surgeon experience; data suggest that rates of ICA injury are inversely proportional to surgeon experience [15]. Objective improvement in endoscopic technique can take up to 100-150 cases, thus when tackling challenging cases, it may be important to recognize one’s technical limitations and ensure the availability of an experienced colleague [3, 16].

## Conclusions

Arterial injuries are the most serious complication of endoscopic skull base surgery, but not all arterial injuries are catastrophic. The critical steps of management are immediate recognition of arterial bleeding, vascular imaging, and vessel sacrifice if necessary. In our case, the patient tolerated carotid artery sacrifice without complication and recovered well without any new neurologic deficit. Emerging endovascular techniques such as endoluminar reconstruction may play an increasing role in the future.
